# Enhancing Polypropylene Biodegradability Through Additive Integration for Sustainable and Reusable Laboratory Applications

**DOI:** 10.3390/polym17050639

**Published:** 2025-02-27

**Authors:** Kanittika Samneingjam, Juthamas Mahajaroensiri, Maysinee Kanathananun, Cristina Velasco Aranda, Mario Muñoz, Somchoke Limwongsaree

**Affiliations:** 1 Innovation and Product Development Center (IPDC), SCG Packaging PLC, Ratchaburi 70110, Thailand; kanittis@scg.com (K.S.); juthamam@scg.com (J.M.); maysinek@scg.com (M.K.); 2 Deltalab Group, 08191 Barcelona, Spain; cristina.velasco@deltalabgroup.com (C.V.A.); mmunoz@deltalabgroup.com (M.M.)

**Keywords:** polypropylene, sustainable labware, autoclave sterilization, cytotoxicity, biodegradability, biodegradable additive

## Abstract

The environmental challenges posed by laboratory plastic waste, particularly single-use items, underscore the urgent need for sustainable alternatives. This study investigated the development of reusable and biodegradable labware, addressing both functional and environmental demands. The content of the biodegradable additive in the polypropylene (PP) varied from 1% to 2% by weight via twin-screw extrusion, followed by injection molding to fabricate test specimens. Three different grades of PP were also compared. Optical, mechanical, and thermal properties were systematically assessed before and after repetitive autoclave sterilization for up to 10 cycles (121 °C, 15 min, 0.11 MPa). Additionally, cytotoxicity following electron beam irradiation (E-Beam 25 and 50 kGy) was evaluated in compliance with ISO 10993-5, alongside biodegradability studies conducted under ASTM D5511 conditions. The results demonstrate that the biodegradable additive stabilized the appearance and enhanced the flexural and impact strengths of PP without compromising thermal stability, particularly after five autoclave cycles. Cytotoxicity assays confirmed the biocompatibility of the additive-modified PP, while biodegradability tests indicated moderate degradation, with 12% biodegradation achieved over 6 months compared to negligible degradation in the negative control. These findings highlight the potential of additive-modified PP as a sustainable solution for reusable labware, balancing durability with improved environmental performance and providing a viable step toward more sustainable laboratory practices.

## 1. Introduction

The demand for laboratory plasticware has been driven by increasing research activities, technological advancements, and expanding healthcare infrastructure. Unlike glassware, plasticware provides durability, easy handling, and a lower upfront cost, meeting the needs of modern laboratory environments regarding affordability and convenience. It has been reported that the research sector globally generates about 5.5 million tons of plastic waste, with single-use plastics (SUPs) making up a significant portion [1,2]. According to environmental concerns, sustainability initiatives, and the need for cost efficiency, there has been a notable shift towards sustainable and reusable labware products, driving demand for reusable and sustainable alternatives. The development of durable and sustainable plastic labware is now gaining more attention from manufacturers in response to environmental concerns, increasing their competitiveness.

Reusable plastic labware can be segmented into non-autoclavable and autoclavable types to serve specific laboratory applications. For example, non-autoclavable plastics such as polystyrene (PS), polyethylene (PE), and certain grades of polypropylene (PP) are commonly used in chemistry laboratories, where heat sterilization is not required but valued for chemical resistance. On the other hand, autoclavable plastics are compulsory for biological, microbiology, clinical, and pharmaceutical works where sterility is vital. Polypropylene (PP) and polycarbonate (PC) can withstand autoclave sterilization, which is a moist heat or steam sterilization. These items must be decontaminated through autoclave sterilization before use, which is carried out at 121 °C for at least 15 min, according to ISO 7218 [3]. PC generally provides superior strength, clarity, and temperature and dimension stability compared to PP [4]. Conversely, PP offers cost and recyclability benefits [5], making it favored for its cost per performance, sustainability, and viability as a reusable labware option for supporting sustainable laboratory practices.

In order to meet its functional requirements, reusable plastic labware must withstand repeated autoclaving cycles without deformation or degradation. Items will be subjected to multiple autoclave sterilizations before being discarded. Autoclave sterilization can cause changes in PP’s properties due to heat, pressure, and moisture. Especially in repetitive autoclave sterilizations, these factors may affect the molecular structure, causing shrinkage and even the physical aging of PP [6,7,8,9]. Materials become more brittle and stiffer and display appearance changes after autoclave sterilization. It has been reported that tensile modulus and elongation at break film increased in PP after steam sterilization. For a PP injection specimen, steam sterilization improved the toughness of the sample due to an annealing effect, resulting in the post-crystallization of PP [10,11].

Apart from superior toughness and heat resistance, durability, adequate chemical resistance, and compliance with safety standards such as those relating to cytotoxicity are also essential to ensure reliable performance and safe usage. In specific applications such as cell culture, tissue culture, and pharmaceutical research, non-cytotoxic labware is a critical requirement to ensure that there is no release of harmful substances from labware that could interfere with the cellular process and results. Cell culture plates, pipettes, centrifuge tubes, and storage containers that are specifically designed for cell work are product examples that generally require non-cytotoxic properties. A cytotoxicity test is, therefore, requested to determine the toxin effect of the material on living cells. There is some evidence strongly suggesting that radiation-induced cytotoxicity results from radiolytically generated reactive species [12]. After the manufacture of sterilized labware, the labware products are commonly treated with irradiation sterilization, such as gamma or electron beam (E-Beam). Hence, it is crucial that the toxicity of the material be assessed after the irradiation sterilization process using ISO 10993-5 [13].

End-of-life challenges are a pressing concern for reusable plastic labware since most of them are non-biodegradable. Recycling is the ideal option. Extended producer responsibility (EPR) programs are initially implemented to manage the collection and recycling of used labware. Unfortunately, insufficient infrastructure and the lack of proper waste collection systems are a reality nowadays. Establishing specialized recycling streams for lab-grade plastics and adopting advanced recycling technologies take time and high investment. Therefore, most plastic wastes end up in landfill or are incinerated, causing adverse effects on the environment. These plastic wastes undergo degradation, resulting in microplastic formation over time [14]. Reducing plastic pollution requires efforts collaborations, and investments to settle sufficient recycling and upcycling facilities and other related infrastructures. However, it may take more than decades to implement holistic waste management worldwide. Therefore, short-term solutions are alternative options to implement.

Eco-friendly biodegradable materials represent one of the short-term options for improving product biodegradability after disposal [15]. The American Society for Testing and Materials (ASTM) defines biodegradable plastic as a degradable plastic that breaks down through the action of naturally occurring microorganisms such as bacteria, fungi, and algae [16]. A clear distinction must be made between biodegradability and biodegradation [17]. Biodegradability refers to the material’s ability to be broken down by living organisms. Biodegradation (also called biotic degradation) describes the degradation process under specific controlled conditions, including temperature, pH, and moisture. Biodegradability is evaluated by measuring microbial degradation through either carbon dioxide and/or methane production, or oxygen consumption, as microorganisms convert the material’s organic carbon into carbon dioxide and/or methane.

Recent research has extensively explored biodegradable polymers as alternatives to disposable plastic labware, with polylactic acid (PLA) emerging as the leading biodegradable candidate [1]. PLA-based tissue culture plates are now commercially available [18] and meet ASTM D6400 [19] standards (commercially compostable). Nevertheless, the widespread adoption of PLA faces several challenges, including higher manufacturing costs than conventional plastics and performance limitations encompassing fragility, limited durability, poor heat resistance, and autoclave incompatibility. Consequently, PLA remains an expensive option with limited applications in reusable labware.

Various testing standards are currently employed for determining plastic biodegradability, with ASTM D6400 [19] and ASTM D5511 [20] being prominent examples that assess biodegradation under distinct environmental conditions, resulting in different test results [21,22]. The ASTM D6400 measures aerobic biodegradation in composting conditions (oxygen-rich), appropriate for commercial composting facilities. The scarcity of commercial composting facilities worldwide means that most plastic waste inevitably ends up in landfills. The ASTM D5511 assesses anaerobic biodegradation settings representing landfill conditions. Therefore, to assess the leakage of plastic waste into nature, ASTM D5511 landfill biodegradation testing provides more relevant data and better simulates conditions that correspond to the majority of plastic waste problems.

In contrast, PP, one of the most commonly used materials in reusable and autoclavable labware applications, can persist in the environment, with an estimated lifetime of up to 450 years before complete biological breakdown [23]. Various approaches have been explored to enhance the degradation of traditional polymers through additive incorporation. Many types of additives have been previously reported and claimed to maintain product functionality while requiring minimal processing modifications. The pro-oxidant additive, for example, which involves metal salts and their complexes, including oxo-degradable plastics that contain pro-oxidant additives (PAC), is developed to stimulate plastic breakdown through oxidation and related processes [24,25]. Unfortunately, they fail to achieve complete degradation and create microplastics under real-world conditions. To overcome this problem, this new biodegradable additive has been introduced as a low-cost solution to handle plastic waste leakage to the environment in the short term without generating microplastic pollution. Incorporating the Biosphere biodegradable additive with a recommended dosage of 1% by weight into the conventional plastic makes the end of life of plastic biodegradable without microplastic formation [26]. It is said to work with all major resin types, including PE, PP, PET, EPS, and EVA [26]. This additive accelerates the biodegradation of these conventional plastics.

Unlike the oxo-degradation additive, the Biosphere additive is biotic and based on enzyme technology. In a microbial-rich environment, the additive promotes microbial colonization and biofilm formation on the product surface, followed by enzyme secretion that activates the biodegradation process, converting the plastic’s carbon sources into gases, biomass, and water [26,27]. Previous studies have reported the effect of using this additive on the mechanical properties of PP [28,29]. In addition, this biodegradable additive is more affordable than the existing biodegradation alternatives [30]. Therefore, it is currently being implemented in commercial products, including labware and consumables, like serological pipets, sample reservoirs, and spatulas. However, the use of this additive in reusable labware is rare, especially in items subjected to multiple autoclave sterilizations before disposal.

To the best of our knowledge, the potential effects of the biodegradable additive on PP’s properties during repeated autoclave cycles and cytotoxicity after irradiation sterilization have never been comprehensively reported. To bridge the gap between durability and sustainability, this study aimed to ensure that PP containing a biodegradable additive can withstand rigorous laboratory use without cytotoxicity while offering an environmentally friendly end-of-life solution. The influence of autoclave cycles combined with the biodegradable additive content (1% and 2% by weight) on the optical and mechanical properties in a homopolymer PP (general-purpose injection molding) was investigated. Subsequently, the 2% additive by weight was explored in two additional PP grades: post-consumer resin (PCR) PP (general-purpose injection molding grade) and the high-melt-flow homopolymer PP (thin wall injection molding grade) to ensure the applicability of the biodegradable additive in various PP grades, including recycled resins. To ensure the safe use of the additive, cytotoxicity testing was performed. Rectangular plastic box samples of homopolymer PP and PP containing 2% additive by weight were injection molded using a commercial injection machine. The samples were then irradiated with an electron beam (E-Beam) at 25 and 50 kGy (to simulate irradiation sterilization) and subsequently evaluated for cytotoxicity following ISO 10993-5 [13] and the MMT cytotoxicity assay with L-929 cell lines. The biodegradation of a plastic box containing 2% additive was investigated under anaerobic conditions of ASTM D5511.

## 2. Materials and Methods

### 2.1. Materials

Three polypropylene injection grades were selected to perform a comparative study, 1100NK (PP homopolymer, Polimaxx, IRPC, Rayong, Thailand), PCRP01JN (SCGC Green Polymer™ PP, SCGC, Bangkok, Thailand), and P901JN (PP homopolymer, SCGC™ PP, Bangkok, Thailand), with melt flow index of 11, 6 and 60 g/10 min (2.16 kg/230 °C), denoted as PP1, PCR PP, and HM PP, respectively. A biodegradable plastic additive, translucent granule masterbatch, was supplied by Biosphere Plastic LLC-USA (grade Biosphere 201, Portland, OR, USA). It has a specific gravity of 1.22–1.26 g/cm^3^ and melt flow index about 45 g/10 min (2.16 kg/230 °C).

### 2.2. Specimen Preparation

In this study, melt blending of PP and additive was conducted using a 20 mm co-rotating twin-screw extruder with 40 L/D ratio (Labtech Engineering Co., Ltd., Samut Prakan, Thailand). To explore the effect of additive content, the additive was varied from 1% to 2% by weight in the PP1, corresponding to PP1 + A1% and PP1 + A2%. The biodegradable additive content was fixed at 2% by weight in the other two, PCR PP and HM PP, to further explore the impact of the additive on different types of PP. The compounded resins of the PP-containing additive were cooled at room temperature for at least 24 h to release stress before characterization and preparation in the next step.

Dog-bone and bar-shaped specimens were prepared using injection molding machine (Nissei Plastic Industrial injection machine, model PS40E5ASE-Japan, Nissei Plastic Industrial Co., Ltd., Nagano, Japan) to obtain samples for mechanical properties testing. The extruder temperature was controlled in range of 198–210 °C, screw speed 150 rpm, and injection pressure 80–105 bar. Obtained specimens were kept in conditions of 23 °C and 50% relative humidity for at least 24 h before testing properties.

**Figure 1:**
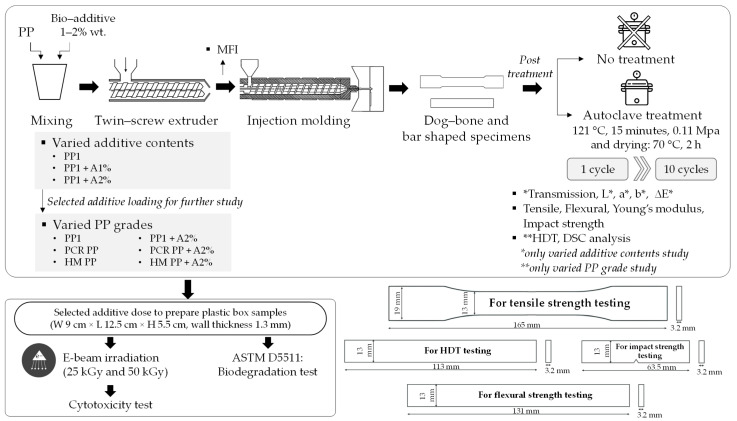
Flow chart for sample preparation, testing processes and dimensions of testing sample.

Rectangular plastic box samples were prepared by Bicappa (Roletto, Turin, Italy) using PP homopolymer and PP containing 2% by weight of the biodegradable additive with a wall thickness of 1.3 mm using a commercial injection molding machine. The samples were denoted as PP box and PP box + A2%, respectively, which were used for cytotoxicity and biodegradation tests. The overall flow of sample preparation and testing is shown in Figure 1.

### 2.3. Autoclave Sterilization Treatment

This work systematically studied the impact of autoclave cycles on the PP and PP-containing biodegradable additive properties. The dog-bone and bar specimens of each formula were subjected to moist heat steam sterilization using an autoclave (TOMY SX 700, Tokyo, Japan) at 121 °C for 15 min under a pressure of 0.11 MPa (15 psi) [3]; then, they were dried in an oven at 70 °C for 2 h before tests or before starting the next autoclave cycle. Process parameters, including autoclaving temperature (121 °C), pressure (15 psi), treatment time (15 min), and drying conditions (70 °C for 2 h), were similarly controlled for all samples. The specimens were exposed from 1 up to 10 consecutive cycles of autoclave sterilization. After treatment for a certain number of autoclaving cycles, samples were taken out to evaluate properties compared with their prior autoclave samples (denoted as Origin).

### 2.4. Characterization and Mechanical Properties Testing

The CIELAB color parameters, L*, a*, and b* (D65/10° illuminant/observer condition), of dog-bone specimens were measured using the Colorimetric Spectrophotometer model 4500L Hunter Lab, Reston, VA, USA. These parameters represent lightness (+L*)/darkness (−L*), red (+a*)/green (−a*), and yellow (+b*)/blue (−b*), respectively. To assess color changes, the total color difference, Delta E* (ΔE*), was calculated using the following equation [29,30]:

(1) $${∆E^{*}=\left[{\left(∆L^{*}\right)}^{2}+{\left(∆a^{*}\right)}^{2}{+\left(∆b^{*}\right)}^{2}\right]}^{1/2}$$

where ∆L*, ∆a*, and ∆b* are the difference of L*, a*, and b* values of sample at Origin (non-autoclave treated) and after autoclave treated for a certain cycle, respectively.

The transmission measurement of dog-bone specimens was tested using Transmission Densitometer model TBX1000, Tobias, Ivyland, PA, USA. % Transmission of specimens was calculated from the equation:

%T = 10^−D^ × 100, (2)

where %T = % light transmission through sample specimen and D = optical density.

Both tensile and flexural tests were conducted using AG X plus Shimadzu Universal Testing Machine (UTM), Kyoto, Japan, with a 10 kN load cell. Tensile properties of PP specimens were examined according to ASTM D638 [31] with a speed of 50 m/min. For flexural properties, the UTM was fitted with a standard three-point bending fixture and samples were flexed until breakage at a rate 13.65 mm/min using a support span of 51.2 mm. Notched izod impact strength of samples was examined at 23 °C according to ASTM D256 [32] using an impact testing machine. The heat distortion temperature (HDT) was determined at 0.45 MPa referring to ASTM D648 [33]. At least 10 specimens were tested and averaged for each test. Dimensions of testing sample for each testing are illustrated in Figure 1.

Melt flow index (MFI) measurements were made under a specific load of 2.16 kg at 230 °C with Melt Flow Tester Zwick/Roell, Ulm, Germany, according to ISO 1133 [34].

Differential scanning calorimetry (DSC) experiments were performed on a Mettler Toledo STARe System DSC3+, Greifensee, Switzerland, in a temperature range of 30–250 °C at a rate of 10 °C/min. The first heating was performed to remove processing history. The first cooling cycle and second heating cycle were used to determine T_c_, T_m_ and %crystallinity.

### 2.5. E-Beam Irradiation and Cytotoxicity Test

The rectangular box samples of PP and PP containing 2% by weight of the additive were then subjected to E-Beam with irradiation doses of 25 and 50 kGy. The dose at 25 kGy is commonly used for sterilizing medical devices, and 50 kGy is the maximum level to account for potential variations in dose distribution. After that, the box samples were finally cut into a standard size of 5 mm × 25 mm and evaluated through cytotoxicity test following ISO 10993-5 [13]; MMT cytotoxicity assay on L-929 cell lines was performed by Thailand Institute of Nuclear Technology (Public Organization), Nakornnayok, Thailand. Cell viability above 70% was considered as a non-cytotoxic material.

### 2.6. Biodegradation Test

The prepared rectangular box of the PP containing 2% by weight of additive (PP box + A2%) was tested under standard high-solids anaerobic digestion conditions of ASTM D5511 by Eden Research Laboratory (Albuquerque, NM, USA). The cumulative gas, carbon dioxide (CO_2_), and methane (CH_4_) evolution during the test was detected from the inoculum, positive control (cellulose), negative control (polyethylene), and the PP box + A2% samples. Regarding the conversion of carbon from the test material to carbon in the gaseous phase, the percent biodegradation was calculated using cumulative CO_2_ and CH_4_ production from each sample after subtracting those produced from the inoculation at 6, 13, 20, and 27 weeks.

## 3. Results and Discussion

### 3.1. Effect of Biodegradable Additive Content and Repeated Autoclaving on PP1 Optical Properties

**Figure 2:**
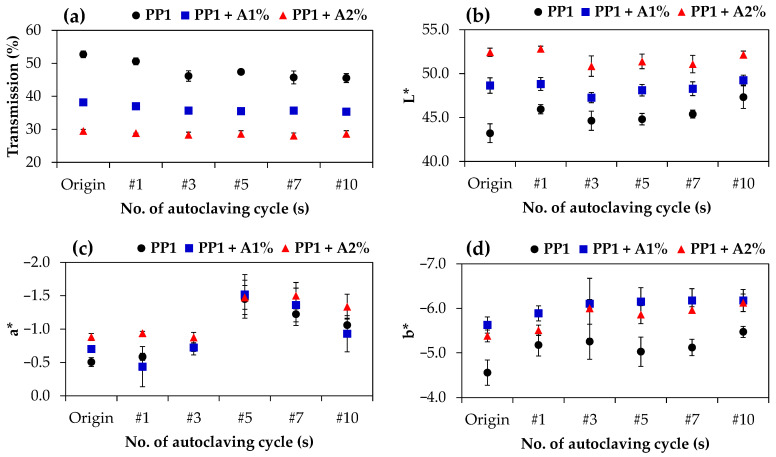
Optical properties of PP1 without additive (PP1, black), PP1 with 1% additive (PP1 + A1%, blue), and PP1 with 2% additive (PP1 + A2%, red) at Origin and after consecutive cycles of autoclave (from 1 cycle to 10 cycles, marked as #1 to #10, respectively): (**a**) transmission, (**b**) L*, (**c**) a*, and (**d**) b*.

Figure 2 illustrates the effect of biodegradable additive content and repeated autoclaving cycles on the optical properties of PP1: %transmission and the CIE color values (L*, a*, and b*). For the neat PP1, an increasing number of autoclaving cycles led to decreased transparency (Figure 2a). A notable reduction in %transmission was observed due to the presence of the additive, particularly at a high loading level of 2% by weight. This reduction, caused by the additive’s translucent appearance, was more pronounced than the influence of repeated autoclaving cycles. While this may limit the implementation of the additive in certain applications requiring high transparency, both additive-containing samples maintained relatively stable transparency despite repeated autoclaving.

**Figure 3:**
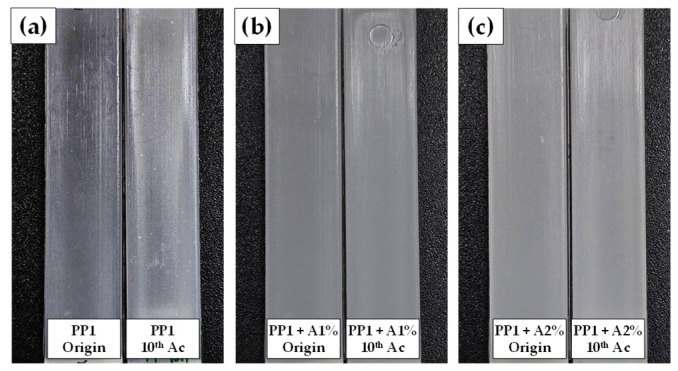
Appearance of Origin samples and samples after 10 cycles of autoclave: (**a**) PP1, (**b**) PP1 + A1%, and (**c**) PP1 + A2%.

A similar trend was observed in lightness (L*) values, where neat PP1 showed a slight increase after one autoclave cycle before stabilizing, while samples with increased additive content displayed noticeably higher L* values that remained constant throughout thermal treatments. Both multiple autoclaving and additive incorporation shifted the sample colors toward green (more negative a*) and blue (more negative b*). To assess the color change magnitude, the Delta E* (ΔE*) values were calculated after autoclaving compared to non-autoclaved originals (non-autoclaved), as shown in Table 1. According to the National Bureau of Standards (NBS), critical remarks of color differences were defined; an ΔE* value of less than 1 was considered not noticeable, values between 1 and 3.3 as perceptible by skilled technicians, and more than 3.3 could be appreciable by a layman [35,36,37]. Although no specific requirement exists for reusable labware, this study adopted ΔE* = 3.3 as the acceptability threshold. Neat PP1 showed noticeable color changes across all autoclave cycles, with ΔE* increasing to 2.8 after one cycle (observable by skilled persons) and reaching 4.2 after 10 cycles, as shown in Figure 3a. In contrast, the PP1-containing additive showed only marginal color changes, with ΔE* values of 0.4 (not noticeable) after one autoclave cycle, remaining below 1.5 and 1.7 throughout multiple treatments, for 1% and 2% additive concentrations, respectively (Figure 3b,c). This level of color change would only be perceivable by experienced workers. This indicates that the additive effectively stabilizes PP color during repeated autoclaving but reduces transparency, which remains stable over multiple cycles.

### 3.2. Effect of Biodegradable Additive Content and Repeated Autoclaving on PP1 Mechanical Properties

**Figure 4:**
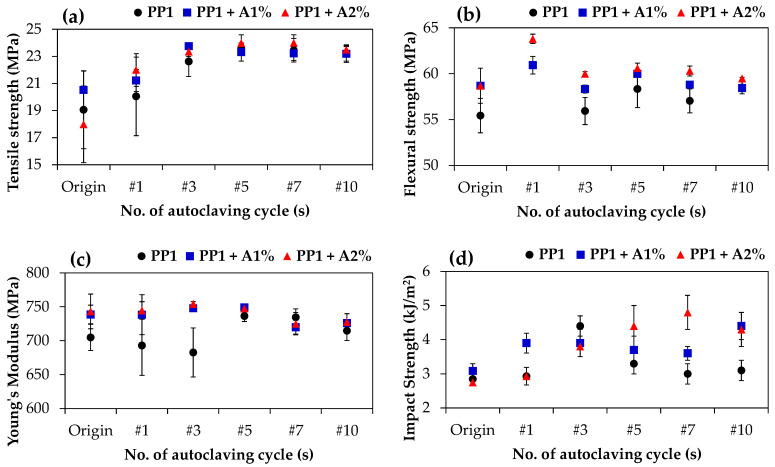
Mechanical properties of PP1 without additive (PP1, black), PP1 with 1% additive (PP1 + A1%, blue), and PP1 with 2% additive (PP1 + A2%, red) at Origin and after consecutive cycles of autoclave (from 1 cycle to 10 cycles, marked as #1 to #10, respectively): (**a**) tensile strength, (**b**) flexural strength, (**c**) Young’s modulus, and (**d**) impact strength.

Figure 4 demonstrates the effects of biodegradable additive content and the number of autoclaving cycles on the mechanical properties of PP1. The tensile strength of the PP1 increased with increasing numbers of autoclaving cycles (Figure 4a), while the flexural strength remained relatively stable with variations (Figure 4b). In the initial untreated state (Origin), incorporating the biodegradable additive marginally enhanced both the tensile and flexural strength (Figure 4a,b) compared to neat PP1. Young’s modulus of PP1 increased after five consecutive cycles of autoclaving, while additive-containing samples consistently exhibited higher Young’s modulus values than the neat PP1 (Figure 4c). This higher Young’s modulus indicates increased material stiffness, suggesting that the biodegradable additive enhances rigidity and improves resistance to deformation in the blends.

In Figure 4d, the impact strength of neat PP1 initially increased but suddenly dropped after five consecutive cycles, while both additive-containing samples revealed substantial improvements in impact strength that became particularly pronounced after autoclaving and remained consistently high throughout thermal treatments, as shown in Figure 4d. In contrast, the pristine PP1 exhibited relatively stable impact strength with only minor variations. In addition, all samples containing the biodegradable additive showed consistent tensile and flexural strength and Young’s modulus throughout 10 consecutive autoclaving cycles, indicating that the additive effectively enhances thermal stability, mitigates thermal degradation, preserves matrix toughness, and improves durability during multiple high-temperature exposures. Based on these findings, the 2% by weight biodegradable additive loading demonstrated optimal overall performance and was, therefore, selected for further exploration with other PP grades in subsequent sections of this study. 

### 3.3. Effect of Biodegradable Additive and Repeated Autoclaving on Different PP Grades

**Figure 5:**
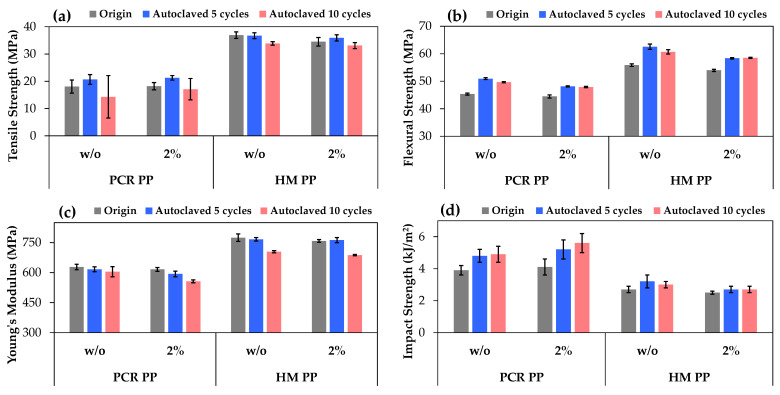
Mechanical properties of two grades of PP (PCR PP and HM PP) without additive (w/o) and PP with additive (2%) at Origin (grey), after autoclaving 5 cycles (blue) and autoclaving 10 cycles (pink): (**a**) tensile strength, (**b**) flexural strength, (**c**) Young’s modulus, and (**d**) impact strength.

To ensure a comprehensive understanding of biodegradable additive behavior in combination with the influence of repeated autoclaving to broaden the additive applicability, two grades of PP, PCR PP and HM PP, were selected for further investigation in this study, with an additive loading of 2% by weight. The mechanical properties of specimens before (Origin) and after 5 and 10 cycles of autoclaving (marked as autoclaved 5 cycles and autoclaved 10 cycles) were systematically investigated, as shown in Figure 5.

The neat HM PP consistently demonstrated superior mechanical properties compared to the PCR PP across all testing conditions, though PCR PP with its lower MFI (Table 2), corresponding to higher molecular weight, showed better impact resistance. In Figure 5d, the impact strength of PCR PP significantly improved after multiple autoclaving cycles, likely because, as a low-melt-flow index polymer, PCR PP under multiple thermal treatments facilitated chain entanglement and molecular rearrangement, enhancing the polymer’s resilience and impact strength [38]. When incorporating a 2% biodegradable additive, varying results were observed across both PP grades. For the PCR PP, the additive generally maintained or partially improved the mechanical properties, while HM PP displayed slight drops in tensile, flexural, and impact strength with the presence of the additive. These findings suggest that the influence and compatibility of the additive would be PP molecularly dependent, which requires further investigation and can be explained by the melt flow index values, as presented in Table 2.

The addition of 2%wt. biodegradable additive increased the MFI for all PP grades, with the magnitude of the effect being grade dependent. Generally, higher MFI values correlate with mechanical properties improvements due to better dispersion and matrix adhesion, leading to tensile and flexural strength enhancements [39]. PP1 + A2% showed slight improved flexural and tensile properties compared to neat PP1 (Figure 4), attributable to higher MFI after additive incorporation (Table 2). The PCR PP displayed constant mechanical properties after additive incorporation, with mostly unchanged MFI values. However, HM PP exhibited the largest MFI increase from 56.49 to 62.80 g/10 min, corresponding to lowering viscosity, which adversely affected the tensile and flexural properties (Figure 5a,b) due to inadequate dispersion and adhesion [39]. This correlation between mechanical properties and the MFI of the PP-containing additive, representing the interaction mechanisms between the additive and specific PP grades, is crucial for tailoring the processing and performance of PP-based materials in industrial applications.

According to multiple autoclaving effects, both PP grades behaved differently. PCR PP remained stable for up to 5 cycles but lost strength at 10 cycles due to some degradation, while HM PP displayed better stability through multiple heat treatments, with only partially reduced properties after 10 cycles, especially in Young’s modulus (Figure 5c). It was reported that autoclave sterilization caused polymer degradation, indicating a slight decrease in average molecular weight and a marginal increase in the polydispersity of the polypropylene after one cycle of autoclaving [40]. Although a single autoclave cycle might slightly degrade the polymers, extended autoclave cycling eventually leads to some structural deterioration and strength loss. Some improvement in flexural and impact properties was observed in both PP grades during initial autoclaving cycles, possibly due to annealing effects.

The synergistic effects of additive incorporation and autoclave treatment resulted in enhanced overall material performance. The presence of a 2% additive by weight helped stabilize the properties of PP during repeating autoclave treatment, suggesting that the additive may create more mobility and facilitate polymer molecular rearrangements during the repeated autoclave cycles. These results indicate that the additive combined with autoclave treatment can effectively modify and enhance PP properties, with the specific effects varying by PP grade and number of treatment cycles.

### 3.4. Effect of Biodegradable Additive and Repeated Autoclaving on Thermal Properties

A comparative study was conducted to examine the thermal properties (HDT testing and DSC analysis) of three PP grades: PP1, PCR PP and HM PP. Test samples were prepared by compounding each PP grade with 2% by weight of additive, followed by injection molding (Figure 1). The injection specimens were exposed for 5 and 10 consecutive autoclave cycles, labelled as autoclaved 5 cycles and autoclaved 10 cycles, respectively. The thermal properties, HDT testing and DSC analysis of treated and untreated (Origin) samples were compared. The thermal properties are presented in Table 3.

From DSC analysis, the crystallization and melting behaviors of all samples are shown as the melting temperature (T_m_), crystallization temperature (T_c_) and the percent crystallinity in Table 3. Each PP grade showed distinct thermal behavior due to differences in MFI and compositions. Generally, a high MFI grade indicates smaller molecular weight, leading to easier chain mobility and, thus, lower T_m_ and T_c_, and vice versa. Even though the HM PP had the highest MFI (~60 g/10 min), it possessed the highest in both T_m_ and T_c_. In addition, the higher T_c_ theoretically correlates with higher crystallinity due to the promotion of the crystalline region formation and rigidity, leading to higher HDT. However, the higher T_c_ does not always improve the crystallinity of the PP [41]. The relationship between MFI, T_m_, T_c_, and HDT, including other mechanical properties, is complex, deviating by several factors, such as the cooling rate, molecular structure, and nucleation growth dynamics, contributed by material formulations and processing conditions. According to the results from this study, the HM PP possessed the highest T_c_ and HDT (120 °C) with a moderate crystallinity (~47%); PP1 displayed the highest crystallinity at about 51% with the lowest T_c_ and HDT (101 °C).

The impact of additive and autoclave treatment on T_m_ was negligible in all PP grades. The additive and autoclaving also did not alter the T_c_ of PP1. The presence of the additive partially decreased the T_c_ in PCR PP and HM PP. The T_c_ of PCR PP containing 2% by weight of additive remained constant throughout the 10 cycles of autoclaving; a drastic drop was observed in HM PP-containing additive after 10 cycles of autoclaving. A reduction in T_c_ could represent the ongoing degradation of material, resulting in lower molecular weight and reduced mechanical properties [42].

It was found that the % crystallinity marginally decreased with additive incorporation or increasing the number of autoclaving cycles, indicating a larger proportion of the amorphous region, which is more capable of absorbing energy, leading to better impact resistance. The PCR PP possessed the lowest crystallinity percentage, representing a larger amount of the amorphous region and the highest initial impact strength among all PP grades in this study. The inclusion of the additive and autoclaving lowered its crystallinity region (higher amorphous phase), resulting in an impact strength improvement (Figure 5d). However, lower crystallinity did not necessarily indicate poorer HDT. This study reaffirmed that the HDT increased after repeated autoclaving cycles. The decrease in the crystallinity at this level may not affect the mobility or cause deformation of the PP sample, which could explain why the HDT can be retained or increased, even after thermal treatments. Though the additive seems to decrease the HDT of PP, the HDT can be recovered after multiple autoclaving cycles. The autoclave treatment, acting as an annealing effect, allows polymer chains to rearrange and increase rigidity, resulting in higher HDT [43].

Therefore, the thermal properties of PP samples containing the additive showed relatively stable characteristics, with negligible variations, and were able to be maintained after repeating autoclave treatment cycles, indicating good thermal stability retention. This study demonstrates that the strategic combination of additive incorporation and autoclave treatment can effectively modify and enhance PP properties. The specific effects vary depending on the PP grade and number of treatment cycles, offering opportunities for tailored material performance in various applications. This comprehensive understanding of property modifications provides valuable insights for optimizing PP formulations and processing conditions for specific end-use requirements.

### 3.5. Cytotoxicity

**Figure 6:**
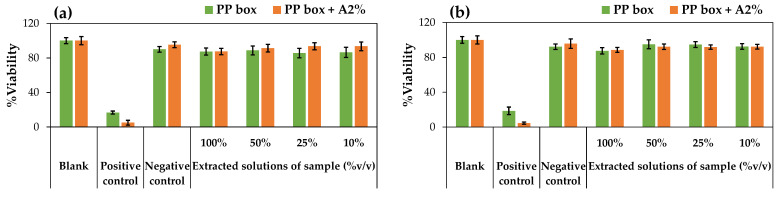
The percentage of cell survival (% viability) after incubation with extracts from PP box and PP box containing 2% by weight of biodegradable additive (PP box + A2%), which were E-Beam irradiation treated at dose of (**a**) 25 kGy (green) and (**b**) 50 kGy (orange).

The cytotoxicity test results of PP box and PP box + A2% samples after E-Beam irradiation at doses of 25 kGy and 50 kGy are presented in Figure 6a,b, respectively. It was found that both samples exhibited cell viability percentages exceeding 70% at all tested extract concentrations (100%, 50%, 75%, 25% and 10%), irrespective of the irradiation dose. In addition, an extract concentration-dependent trend was observed, with cell survival percentages increasing as the extract concentration decreased. This suggests that higher extract concentrations lead to a more pronounced potential influence of any leachable compounds on cell viability, though still within non-cytotoxic levels. The dilution effect observed at lower concentrations likely reduces the impact of these substances, thereby enhancing cell viability. These results confirm that both PP box and PP box + A2% samples after E-Beam irradiation are non-cytotoxic materials. In the future, the leachable substances or degradation byproducts generated during irradiation should be further characterized to assess their long-term effects on biocompatibility.

### 3.6. Biodegradation

Biodegradation additives were invented to accelerate the biodegradation of conventional plastics while avoiding microplastic formation [26]. Unfortunately, the exact compositions of the additive have not been reported. According to the product safety data sheet, the additive is primarily composed of hydrophilic components, such as aliphatic-aromatic ester, polylactide, organoleptic, monosaccharides, and aldohexose [44]. In a microbe-rich environment, these components allow microorganisms to colonize the plastic surface and form biofilms, leading to enzyme secretion, which further breaks down polymer chains and enables faster degradation. As a result, smaller molecules are then consumed by microbes for growth and reproduction through the biotic degradation pathway (hydrolysis, acetogenesis, and methanogenesis), ultimately converting the plastic into final products, like CO_2_, CH_4_, biomass, and water.

**Figure 7:**
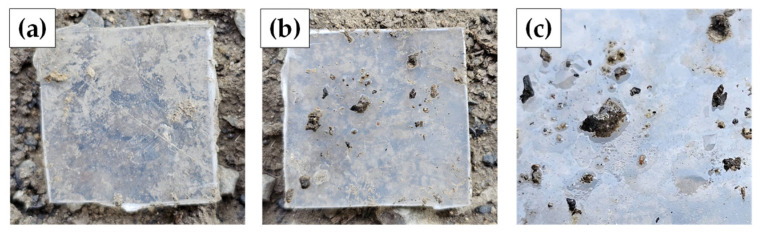
Appearance of samples from a soil burial test for 4 months: (**a**) PP, (**b**,**c**) PP + A2%.

To confirm biofilm formation, a soil burial test was conducted in a grass field using two types of plastic box samples: PP box and PP box + A2%. The samples were cut into 2.5 cm × 2.5 cm squares and buried 30 cm deep for four months. After excavation, visual examination, as illustrated in Figure 7, revealed notable biofilm formation on the surface of PP box + A2% (Figure 7b,c), whereas the PP sample showed no visible alterations (Figure 7a). These results indicate that the additive in the PP box + A2% effectively promotes biofilm formation by attracting environmental microorganisms, potentially facilitating the biodegradation process.

**Figure 8:**
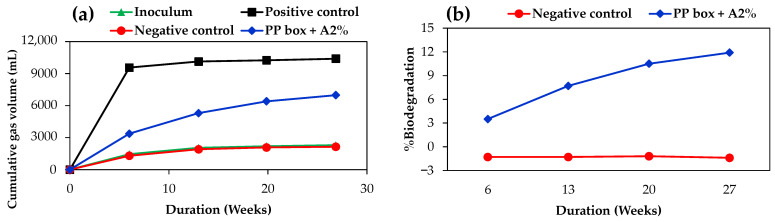
ASTM D5511 biodegradation results: (**a**) cumulative gas volume and (**b**) %biodegradation over 27 weeks. %Biodegradation of the positive control was in the range of 78.7 ± 0.3% during the testing period.

Figure 8 shows the biodegradation results under high-solids anaerobic biodegradation conditions following ASTM D5511. In Figure 8a, the cumulative gas volume, measured in milliliters (mL), illustrates the evolution of CO_2_ and CH_4_ over 27 weeks for the inoculum, positive control (cellulose), negative control (polyethylene, PE), and the PP box + A2%. As expected, the positive control produced the highest cumulative gas volume, reflecting the biodegradation nature of cellulose. In contrast, the negative control, consisting of PE, displayed negligible gas production, even lower than the inoculum, reaffirming its resistance to microbial degradation. The PP box + A2% exhibited moderate but steadily increasing cumulative gas production, indicating the conversion of the carbon in the PP material to gaseous CO_2_ and CH_4_, facilitated by the biodegradable additive. This is further supported by the significantly higher cumulative gas volume and percent biodegradation compared to the negative control (Figure 8b), confirming that the biodegradable additive enhances the biodegradation potential of PP material under the tested conditions.

In Figure 8b, the biodegradation percentage is shown as a function of time for the negative control and the PP box + A2%. The positive control exhibited a biodegradation percentage of 78.7 ± 0.3% throughout the testing period, while the negative control remained nearly constant below zero, as its cumulative gas volume was lower than that of the inoculum (Figure 8a). The PP box + A2% displayed a gradual increase in biodegradation percentage, reaching approximately 12% at 27 weeks with a continuous upward trend, indicating enhanced biodegradation due to the additive. Although the sample showed relatively low biodegradation results within the testing period, there is no specific threshold of biodegradation percentage for ASTM D5511. This differs from the ASTM D6400, which requires more than 90% biodegradation within 180 days. Furthermore, the biodegradation percentages vary depending on the testing conditions. For example, a previous study reported that PLA completely degraded in 49 days under ASTM D6400 testing conditions, whereas under ASTM D5511 conditions, it achieved only 60% biodegradation within 40 days [21,22].

Compared to previous studies under the ASTM D5511 conditions, a conventional PP straw sample displayed only 2.7% biodegradation over 924 days, whereas a PP straw-containing additive sample achieved 33.7% biodegradation, highlighting the inherently slow biodegradation of PP. Moreover, the rates of biodegradation are also thickness-dependent, with thicker samples degrading more slowly. However, the thickness was not explicitly reported, and the slow biodegradation rates in previous studies suggest the use of thick samples with low additive loading [26]. In contrast, another study found that 0.3 mm thick PE with 1% additive showed approximately 14% biodegradation in just 18 days [30]. The relatively low biodegradation rate observed in this study is likely due to the sample’s greater thickness (1.3 mm). Nevertheless, the incorporation of 2% biodegradable additive significantly enhanced PP’s biodegradation compared to conventional PP, demonstrating its effectiveness under anaerobic digestion (landfill) conditions. Extended testing is expected to further increase the biodegradation percentage.

This finding highlights the need for the further optimization of additive formulations and concentrations to achieve greater biodegradation efficiency. Additionally, this study emphasizes the importance of testing under real-world conditions to validate the performance of such materials in practical applications. Overall, the addition of biodegradable additives presents a promising approach to improving the environmental performance of traditional plastics, though further research and development are required to enhance their degradation rates.

## 4. Conclusions

This study investigated the effects of incorporating 1–2% by weight of biodegradable additive into polypropylene (PP), demonstrating enhanced mechanical properties and thermal stability during multiple autoclave cycles while enabling end-of-life biodegradation capabilities. At 2% loading, the modified PP exhibited stabilized mechanical properties through 10 consecutive autoclave cycles, showing improved impact strength and Young’s modulus compared to neat PP. Although the additive reduced transparency, it effectively stabilized color changes, maintaining professional-level color consistency throughout multiple uses. The additive’s effectiveness varied across PP grades, with optimal results in general-purpose PP compared to PCR and high-melt-flow grades, while cytotoxicity testing confirmed the non-toxic characteristics after E-beam irradiation at both 25 and 50 kGy doses. The soil burial test visually confirmed biofilm formation, demonstrating that that the additive successfully promotes microbial colonization. This formation of biofilms facilitated the biodegradation process, enhancing the breakdown of the PP matrix and achieving 12% biodegradation over 27 weeks under anaerobic conditions (ASTM D5511). However, the sample thickness limited the degradation rates. These findings demonstrate the potential for biodegradable additives to enhance PP’s sustainability while maintaining performance in laboratory applications. Future research should address optimizing additive concentrations and processing conditions for different PP grades, evaluating long-term biocompatibility effects, particularly regarding leachables and extractables after repeated autoclaving, and investigating extended biodegradation behavior under varied environmental conditions to ensure broader applicability and practical implementation in research and industrial settings.

## Figures and Tables

**Table 1:** ΔE* values of PP1, PP1 + A1%, and PP1 + A2% after different autoclaving cycles compared to their origin color.

Sample	ΔE* After Autoclave Treatment(s)				
	1 Cycle	3 Cycles	5 Cycles	7 Cycles	10 Cycles
PP1	2.8	1.6	1.9	2.4	4.2
PP1 + A1%	0.4	1.5	1.1	0.9	0.9
PP1 + A2%	0.4	1.7	1.3	1.6	0.9

**Table 2:** Melt flow index (MFI) of different PP grades (without additive) and PP with 2% wt. additive.

MFI (g/10 min)	Without Additive	With 2% by Weight of Additive
PP1	10.36 ± 0.07	11.23 ± 0.05
PCR PP	6.49 ± 0.03	6.54 ± 0.03
HM PP	56.49 ± 0.43	62.80 ± 0.66

**Table 3:** HDT and DSC results of PP blends with biodegradable additive.

Sample		HDT (°C)		T_m_ (°C)		T_c_ (°C)		% Crystallinity	
		PP	PP + A2%	PP	PP + A2%	PP	PP + A2%	PP	PP + A2%
PP1	Origin	101	103	165.8	166.7	120.8	120.5	50.6	50.8
	Autoclaved 5 cycles	122	125	166.9	165.7	120.0	120.4	47.8	48.2
	Autoclaved 10 cycles	127	125	166.4	167.0	120.2	120.6	49.2	48.8
PCR PP	Origin	104	99	162.0	161.5	124.7	122.8	41.2	42.2
	Autoclaved 5 cycles	119	118	162.2	162.6	124.2	122.0	44.2	40.0
	Autoclaved 10 cycles	119	122	161.6	161.8	124.2	122.2	43.1	40.6
HM PP	Origin	120	117	159.3	158.8	129.8	128.2	47.4	46.9
	Autoclaved 5 cycles	129	127	159.3	158.8	129.3	127.4	47.0	45.1
	Autoclaved 10 cycles	130	127	159.1	157.2	129.1	120.3	45.1	44.3

## Data Availability

Data supporting this study are included within the article.
